# Utidelone-associated chemotherapy-induced peripheral neuropathy: an evidence-stratified narrative review of mechanisms, risk identification, and management strategies

**DOI:** 10.3389/fonc.2026.1927392

**Published:** 2026-08-26

**Authors:** Sili Li, Lifeng Lei, Yi Luo

**Affiliations:** Department of Thyroid and Breast Surgery, Nanchong Central Hospital Affiliated with North Sichuan Medical College, Nanchong, Sichuan, China

**Keywords:** chemotherapy-induced peripheral neuropathy, dose modification, epothilone, metastatic breast cancer, neurotoxicity, patient-reported outcomes, supportive care, utidelone

## Abstract

Utidelone is a genetically engineered epothilone analog and microtubule-stabilizing agent developed in China. It demonstrates antitumor activity in recurrent or metastatic breast cancer after anthracycline and taxane failure. In the pivotal phase III BG01-1323L trial, utidelone plus capecitabine improved progression-free and overall survival versus capecitabine alone; however, peripheral neuropathy occurred in 59% of combination patients, including grade 3 in 22%, versus 8% and less than 1%, respectively, with capecitabine alone. Clinically, neuropathy may impair fine motor function, gait, sleep, and other daily activities, effects not fully captured by toxicity grades alone. Prospective and real-world studies confirm that peripheral neuropathy remains the principal treatment-limiting toxicity of utidelone. Utidelone-specific mechanistic evidence is emerging but remains limited. A 2025 mouse study linked utidelone-induced mechanical and cold allodynia to TRPA1 upregulation and oxidative stress in dorsal root ganglia. Beyond this single preclinical finding, proposed mechanisms--including altered microtubule dynamics, impaired axonal transport, mitochondrial dysfunction, and neuroinflammation--are largely extrapolated from taxane and platinum research. Their relevance to utidelone-induced numbness or persistent deficits in humans remains speculative. Neither ASCO nor ESMO-EONS-EANO recommends routine pharmacologic prophylaxis for chemotherapy-induced peripheral neuropathy (CIPN). Duloxetine has the strongest evidence base for treating established painful CIPN but has not been evaluated specifically for utidelone-associated neuropathy. A small randomized trial found that electroacupuncture produced greater symptomatic improvement than mecobalamin in patients with utidelone-associated neuropathy, although larger confirmatory trials are needed. Evidence supporting monosialotetrahexosylganglioside (GM1), exercise, acupuncture, cryotherapy, compression therapy, or neuromodulation remains preliminary, indirect, or context-dependent. We distinguish utidelone-specific evidence from evidence extrapolated from taxane-, platinum-, and mixed-CIPN populations, because broader CIPN findings are frequently applied to patients receiving utidelone despite uncertain direct applicability. On the basis of available evidence and established CIPN principles, we propose a practical framework integrating pretreatment neurologic and risk assessment, cycle-by-cycle monitoring, symptom-directed supportive care, fall prevention, rehabilitation, and individualized modification of anticancer treatment. Prospective studies are needed to define the onset, cumulative dose–toxicity relationship, persistence, recovery trajectory, and modifiable risk factors of utidelone-associated neuropathy.

## 1 Introduction

Utidelone is a cytotoxic agent with demonstrated progression-free and overall survival benefits in patients with metastatic breast cancer previously treated with anthracyclines and taxanes (1, 2). However, peripheral neuropathy is a major treatment-limiting toxicity. In the phase III BG01-1323L trial, peripheral neuropathy occurred in 59% of patients receiving utidelone plus capecitabine, including grade 3 events in 22% (1). This review separates direct utidelone evidence from extrapolation.

Utidelone is a genetically engineered epothilone analog that promotes tubulin polymerization and microtubule stabilization (1, 3). Its molecular structure and resistance profile differ from those of taxanes, and preclinical and early clinical findings suggest that it may retain antitumor activity in some taxane-resistant tumors, including tumors with P-glycoprotein-mediated drug efflux (3). Whether these pharmacologic differences result in a distinct neurotoxicity profile remains unknown. No prospective head-to-head study has compared the onset, severity, phenotype, cumulative dose dependence, persistence, or recovery of utidelone-associated neuropathy with neuropathy caused by taxanes or other microtubule-targeting agents.

Clinical efficacy was established in the phase III BG01-1323L trial, in which utidelone plus capecitabine improved progression-free survival and overall survival compared with capecitabine alone in heavily pretreated metastatic breast cancer (1, 2). Subsequent observational studies have reported antitumor activity in earlier treatment lines and in comparison with eribulin, although their retrospective designs limit causal interpretation (4, 5). The toxicity profile is dominated by peripheral neuropathy. In BG01-1323L, the marked disparity between the combination group and the capecitabine-alone arm indicated that neurotoxicity is driven primarily by utidelone (1). A 270-patient multicenter real-world study confirmed a high neuropathy burden and identified two factors associated with severe toxicity: unresolved neuropathy from previous treatment and the conventional days 1–5 administration schedule (6). The toxicity is not limited to breast cancer. In a phase II study of refractory soft-tissue sarcoma, grade ≥3 peripheral neuropathy occurred in 11% of patients receiving utidelone monotherapy (7).

A prospective multicenter study evaluating a 120-hour continuous-infusion schedule reported a lower rate of grade ≥3 peripheral neuropathy, suggesting that the administration schedule may influence tolerability (8). This schedule looks promising, but the study was nonrandomized and included heterogeneous solid tumors, so it cannot establish superiority over the conventional days 1–5 schedule.

Chemotherapy-induced peripheral neuropathy (CIPN) most commonly manifests as distal symmetric numbness, tingling, paresthesia, burning or electric pain, sensory loss, allodynia, reduced reflexes, impaired dexterity, balance disturbance, or gait instability (9, 10). A systematic review estimated that CIPN occurs in approximately 68% of patients within the first month after neurotoxic chemotherapy, 60% at 3 months, and 30% at 6 months or later, although estimates vary considerably according to the causative drug, cumulative exposure, assessment method, and patient population (11). CIPN may lead to dose interruption, dose reduction, or treatment discontinuation. It may also persist after chemotherapy has ended and adversely affect mobility, independence, sleep, mood, social participation, and quality of life (12, 13). Persistent CIPN has been associated with falls, balance impairment, and functional disability among cancer survivors (14, 15).

Utidelone is commonly administered after taxane exposure. Some patients may therefore begin treatment with persistent symptoms or subclinical nerve injury related to previous therapy. This clinical context raises specific questions regarding baseline assessment, cumulative neurotoxicity, treatment monitoring, dose modification, supportive care, and recovery.

This narrative review aims to summarize the clinical burden of utidelone-associated peripheral neuropathy; distinguish direct utidelone evidence from extrapolated CIPN evidence; evaluate assessment, risk identification, prevention, and management strategies; and propose an evidence-calibrated clinical workflow.

## 2 Literature search and evidence classification

### 2.1 Search strategy

A targeted narrative literature search was performed using PubMed/MEDLINE, Embase, Web of Science, the Cochrane Library, and major Chinese biomedical databases. Publications available through July 31, 2026, were considered. Search terms included combinations of:”utidelone”; “epothilone”; “metastatic breast cancer”; “chemotherapy-induced peripheral neuropathy”; “peripheral neurotoxicity”; “neuropathic pain”; “small-fiber neuropathy”; “risk factor”; “patient-reported outcome”; “vibration perception threshold”; “dose modification”;”duloxetine”;”GM1” or”monosialotetrahexosylganglioside”;”mecobalamin”;”acupuncture” or “electroacupuncture”; “exercise” or “rehabilitation”; “cryotherapy”; “compression therapy”; and “Scrambler therapy.”

Priority was given to utidelone-specific prospective and retrospective studies, randomized trials, international clinical practice guidelines, systematic reviews, meta-analyses, and mechanistic studies directly relevant to CIPN. Since direct evidence regarding utidelone-associated CIPN remains limited to date, this review incorporates indirect evidence from taxanes, platinum compounds, vinca alkaloids and other microtubule-targeting agents where appropriate, and explicitly designates such findings as extrapolated evidence.

Because this was a narrative review, the Preferred Reporting Items for Systematic Reviews and Meta-Analyses framework was not applied, and the review did not include protocol registration, duplicate screening, risk-of-bias assessment, quantitative meta-analysis, or Grading of Recommendations Assessment, Development and Evaluation certainty ratings.

### 2.2 Evidence classification

We sorted evidence into five distinct groups based on how relevant each study was to utidelone-associated peripheral neuropathy, instead of relying on study design, methodological rigor, or overall certainty. We did not perform formal structured risk-of-bias evaluation for this review, nor did we use the GRADE framework. For this reason, these groupings cannot serve as official certainty ratings. Any conclusions built on extrapolated evidence or clinical suggestions put forward by the authors are clearly noted within the text.

The five groups are as follows:

1. Direct comparative human utidelone evidence: randomized trials and other studies with concurrent control groups that enrolled patients given utidelone;
2. Direct non-comparative human utidelone evidence: prospective single-arm trials, retrospective analyses, real-world datasets, and secondary analyses among utidelone-treated patients;
3. Direct utidelone-specific preclinical evidence: animal, cell-, or tissue-level laboratory work assessing neurotoxicity caused directly by utidelone;
4. Extrapolated CIPN evidence: data obtained from taxanes, platinum-based chemotherapies, other microtubule-targeting agents, or mixed patient cohorts with chemotherapy-induced peripheral neuropathy;
5. Author-proposed clinical practice: clinical workflows and management strategies assembled by the authors, combining available utidelone-specific data, established international CIPN guidelines, and relevant extrapolated evidence.

## 3 Clinical features and burden of utidelone-associated CIPN

### 3.1 Incidence and severity

The BG01-1323L trial provides the strongest comparative evidence on utidelone-associated peripheral neuropathy. Among 267 patients assigned to utidelone plus capecitabine, peripheral neuropathy of any grade occurred in 158 patients (59%), including grade 3 events in 58 patients (22%); no grade 4 neuropathy was reported. In the capecitabine-alone group, peripheral neuropathy occurred in 11 of 130 patients (8%), and grade 3 events occurred in fewer than 1% of patients (1). This difference suggests that neurotoxicity is primarily associated with utidelone exposure.

In a study of 270 pretreated patients with metastatic breast cancer, peripheral neuropathy occurred in 55.2% of patients (6). Unresolved neuropathy from previous therapy was associated with grade ≥3 neuropathy (severe-grade events per Common Terminology Criteria for Adverse Events [CTCAE]) during utidelone treatment. The conventional days 1–5 administration schedule was also associated with severe neuropathy; however, this retrospective study design cannot establish definitive causal relationships (6).

Additional studies suggest that clinically significant neuropathy is not confined to metastatic breast cancer. In a prospective single-arm study of patients with heterogeneous advanced solid tumors who received a 120-hour continuous utidelone infusion, grade ≥3 peripheral neuropathy occurred in 4% of participants (8). In the UTISARC phase II study of refractory soft-tissue sarcoma, peripheral neuropathy was the most frequent grade ≥3 adverse event, occurring in 11% of patients (7). Neither study was designed to compare the neurotoxicity of alternative dosing schedules or regimens.

**Table 1** summarizes direct human studies reporting peripheral neuropathy outcomes during utidelone treatment.

**Table 1:** Direct clinical evidence on utidelone-associated peripheral neuropathy: group 1 (comparative human) and group 2 (non-comparative human) studies.

Design and population	Regimen or schedule	Main neuropathy findings	Key limitation	Ref
Randomized phase III trial; metastatic breast cancer	Utidelone plus capecitabine versus capecitabine alone; n = 267 versus 130	Any grade: 59% versus 8%; grade 3: 22% versus <1%; no grade 4 events with utidelone	Limited baseline neurologic, patient-reported, and long-term recovery data	(1)
Retrospective multicenter study; 270 patients with pretreated metastatic breast cancer	Utidelone-based treatment, including the conventional days 1–5 schedule	Any grade: 55.2%; unresolved neuropathy from previous therapy and the conventional schedule were associated with severe neuropathy	Retrospective design and potential confounding; no causal inference regarding schedule	(6)
Prospective multicenter single-arm study; heterogeneous advanced solid tumors	Utidelone-based therapy using 120-hour continuous infusion	Grade ≥3: 4%	No randomized schedule comparison; heterogeneous tumors and regimens	(8)
Single-arm phase II study; refractory soft-tissue sarcoma	Utidelone monotherapy	Grade ≥3: 11%	Small, single-arm soft-tissue sarcoma study; limited phenotype and recovery data	(7)

All studies listed represent Group 1 (randomized trials with concurrent controls) or Group 2 (prospective single-arm, retrospective, or real-world) evidence as defined in Section 2.2. Cross-study comparisons are limited by differences in study populations, prior neurotoxic exposure, baseline neuropathy, regimen, assessment method, and follow-up.

Across the available studies, neuropathy emerging during treatment has not been clearly distinguished from pre-existing or residual neuropathy present prior to therapy. Data also remain incomplete on median time to onset, cumulative-dose dependence, painful versus numbness-dominant phenotypes, dose modification attributable specifically to neuropathy, reversibility after treatment modification, and persistent neuropathy after treatment completion.

These incidence figures have immediate clinical implications. In BG01-1323L, approximately one in five patients receiving utidelone plus capecitabine developed grade 3 peripheral neuropathy, representing severe toxicity with the potential to impair self-care and prompt treatment interruption, dose reduction, or discontinuation (1). Beyond clinician-assigned toxicity grades, CIPN may compromise hand dexterity, gait, balance, independence, and quality of life and is associated with an increased risk of falls and functional disability (12–15). The clinical significance of utidelone-associated neuropathy should therefore be evaluated not only by its incidence but also by its effects on daily function, treatment continuity, and patient preferences. These considerations support pretreatment neurological documentation, early symptom reporting, and cycle-by-cycle assessment as integral components of routine care rather than optional measures (16, 17).

### 3.2 Clinical phenotype

No prospective comparative study has established a neurologic phenotype unique to utidelone. Available reports are broadly consistent with a length-dependent sensory neuropathy.

Although utidelone-specific phenotyping is unavailable, the general CIPN literature describes a characteristic set of sensory disturbances, including bilateral distal numbness, paresthesias, burning or shooting pain, mechanical hypersensitivity, cold-evoked discomfort or allodynia, impaired vibration or position sense, diminished grip strength or manual dexterity, gait instability, impaired balance, and falls (9, 10).

Motor findings are generally less prominent than sensory symptoms, although functional motor impairment may emerge secondary to sensory loss, pain, proprioceptive deficits, deconditioning, or direct motor nerve injury.

### 3.3 Small-fiber injury

Small-fiber involvement may contribute to burning pain, thermal abnormalities, and allodynia in patients with CIPN. Corneal confocal microscopy has been investigated as a noninvasive method for detecting small-fiber injury and nerve regeneration in patients receiving neurotoxic chemotherapy (18).

These findings are relevant to CIPN generally but have not been validated in utidelone-treated populations. Normal nerve conduction studies therefore do not exclude clinically important small-fiber or pain-predominant neuropathy.

### 3.4 Functional and quality-of-life burden

Clinician-assigned toxicity grades may not fully capture the burden of neuropathy experienced by patients. Physician assessments and patient reports of CIPN often differ, and clinicians may underestimate numbness, tingling, pain, fine motor impairment, or functional difficulties in daily life (19, 20).

Persistent CIPN is associated with impaired quality of life (13), increased falls and disability (14), and measurable balance and functional deficits (15). These consequences are especially important among patients with metastatic breast cancer, in whom neuropathy may coexist with fatigue, anemia, pain, sarcopenia, bone metastases, and treatment-related deconditioning.

## 4 Assessment and monitoring

### 4.1 Clinician grading

The Common Terminology Criteria for Adverse Events (CTCAE) provides a standardized framework for grading peripheral sensory and motor neuropathy. This grading system is useful for safety reporting, treatment comparison, and dose-modification decisions.

However, clinician grading alone may be insensitive to early symptoms, intermittent abnormalities, neuropathic pain, and functional interference. Prospective research has shown that the apparent incidence of CIPN varies depending on the assessment method used (20).

CTCAE grading should therefore be combined with patient-reported outcomes and focused neurologic assessment.

### 4.2 Patient-reported outcomes

Standardized patient-reported outcomes are important in clinical trials and routine monitoring. Recommendations for trial design emphasize the use of reliable, responsive, and clinically meaningful CIPN outcome measures (21).

Validated instruments include: the European Organization for Research and Treatment of Cancer Quality of Life Questionnaire–Chemotherapy-Induced Peripheral Neuropathy 20-item module (EORTC QLQ-CIPN20) (22); the Functional Assessment of Cancer Therapy/Gynecologic Oncology Group–Neurotoxicity questionnaire (FACT/GOG-Ntx) (23); the National Cancer Institute Patient-Reported Outcomes Version of the Common Terminology Criteria for Adverse Events (PRO-CTCAE) (24); standardized clinician-rated and composite measures evaluated in the Chemotherapy-Induced Peripheral Neuropathy Outcome Measures Standardization Study (25), and The EORTC QLQ-CIPN20 has demonstrated acceptable reliability and validity for patient-reported sensory, motor, and autonomic symptoms (26).

At each cycle, ask about the classic glove-and-stocking symptoms: numbness, tingling, burning or shooting pain. Do not forget cold allodynia. Also inquire about gait stability, falls, and hand function, as these are what patients actually care about. Clinicians should also screen for reduced sensory perception, trouble gripping or handling daily objects, and functional impairment affecting fine motor tasks such as writing. Additional important indicators include unsteady walking patterns, fall or near-fall experiences, sleep disturbances secondary to neuropathic discomfort, and reduced independence in routine daily activities. Serial assessment of change from baseline is more informative than a single isolated score.

### 4.3 Neurologic assessment and objective testing

Baseline and follow-up neurologic examinations may include assessment of light-touch sensation, pinprick sensation, vibration sensation, proprioception, deep tendon reflexes, distal upper- and lower-extremity strength, grip function, gait, tandem walking and balance. These evaluations should be tailored to the specific clinical context and the patient’s baseline risk. Nerve conduction studies can identify large-fiber sensory or motor abnormalities and help differentiate CIPN from other neuromuscular disorders. They are most helpful for clinical pictures that are severe, asymmetric, proximal, rapidly progressive, predominantly motor, or diagnostically uncertain. Even so, normal nerve conduction findings do not rule out small-fiber neuropathy.

A 2026 systematic review and meta-analysis of 31 studies involving 1,635 participants found that vibration perception thresholds consistently worsened after neurotoxic chemotherapy in the hands and feet (27). Vibration testing may therefore offer a practical objective measure of large-fiber dysfunction. However, diagnostic thresholds, predictive value, and utility for utidelone-specific monitoring have not been validated.

### 4.4 Guideline-based assessment principles

The ESMO-EONS-EANO guideline recommends regular assessment of neurologic symptoms and function, together with evaluation for alternative diagnoses, in patients receiving potentially neurotoxic treatment (16). The ASCO guideline similarly emphasizes clinical monitoring and consideration of treatment modification when neuropathy becomes intolerable or impairs function (17). Neither guideline identifies a single assessment instrument as sufficient for all purposes. A combined approach using clinician grading, patient-reported symptoms, and functional assessment is therefore reasonable.

## 5 Mechanistic basis

**Figure 1:**
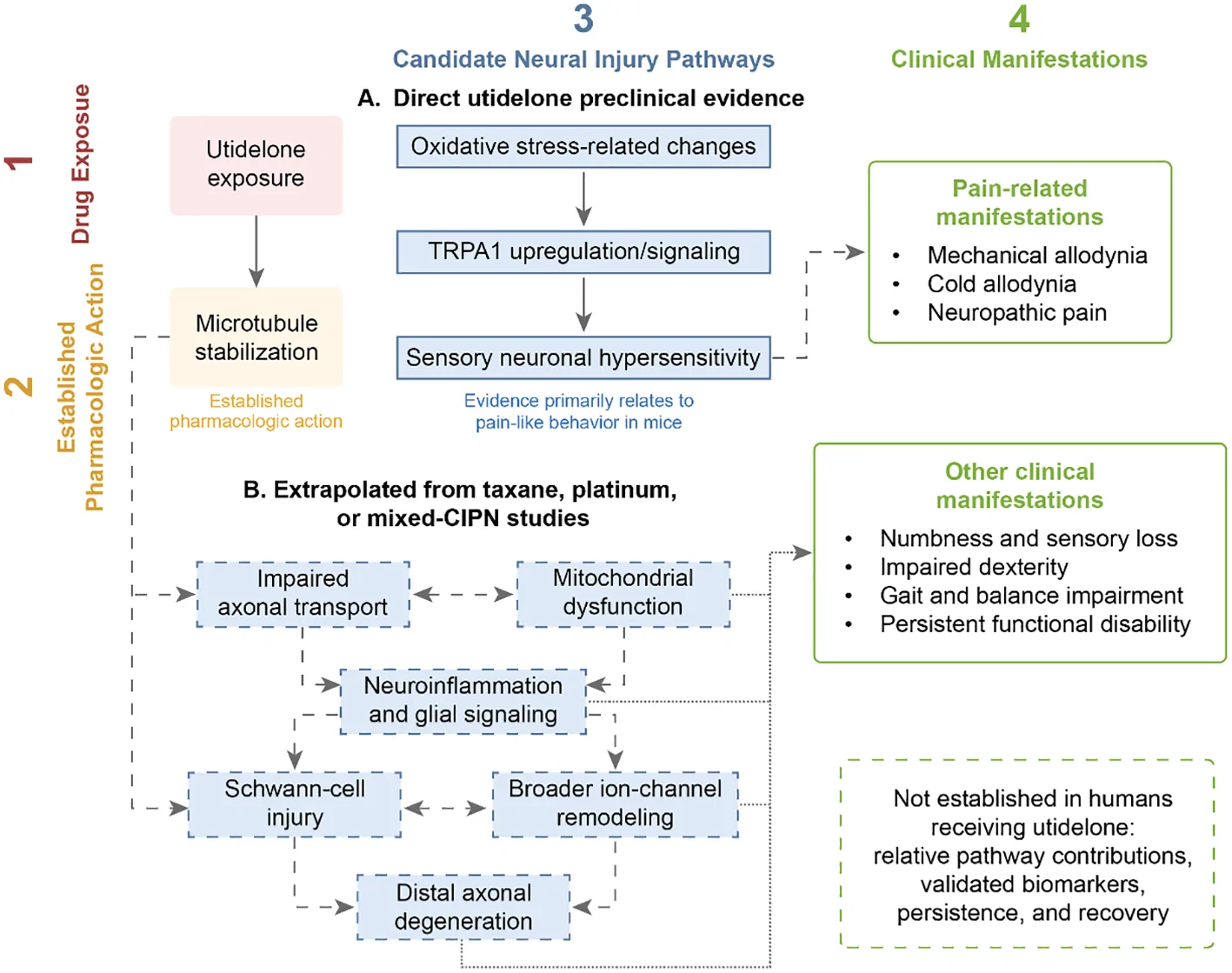
Evidence-stratified framework for the pathogenesis of utidelone-associated peripheral neuropathy. Established pharmacology: Utidelone promotes tubulin polymerization and microtubule stabilization (1, 3). Group 3 evidence: A mouse study linked oxidative stress-related changes and increased TRPA1 expression to mechanical and cold allodynia after utidelone exposure (28). Group 4 (extrapolated): Impaired axonal transport, mitochondrial dysfunction, neuroinflammation, Schwann cell injury, broader ion-channel remodeling, and distal axonal degeneration are supported mainly by taxane, platinum, and mixed CIPN studies (28–41). Group 5 (author-proposed): Dotted arrows indicate mechanistic links that remain unvalidated. Solid arrows, Group 3; dashed arrows, Group 4; dotted arrows, Group 5. The contributions of these pathways to numbness, functional impairment, persistent neuropathy, and neurologic recovery in humans receiving utidelone remain unknown.

The mechanistic framework in **Figure 1** reflects the directness of the supporting evidence and does not present all proposed pathways as equally established. Microtubule stabilization is the established pharmacologic action of utidelone (1, 3). Direct preclinical data are limited to a single 2025 mouse study linking utidelone to mechanical and cold allodynia via TRPA1 upregulation and oxidative stress in the dorsal root ganglia (28). This finding explains pain-like behavior but does not address numbness, large-fiber loss, or persistent neuropathy in humans. Beyond the TRPA1 finding, the remaining mechanisms are educated guesses borrowed from taxane and platinum research. Impaired axonal transport, mitochondrial injury, neuroinflammation, Schwann cell damage—each is plausible, and together they form a coherent pathophysiological story. But coherence is not proof. Whether they explain the numbness-dominant phenotype, the gait instability, or the persistent deficits that clinicians actually see in utidelone patients remains entirely open (28–41).

### 5.1 Microtubule stabilization and axonal transport

Utidelone exerts antitumor activity by promoting tubulin polymerization and microtubule stabilization (1, 3). Microtubules are also essential components of neuronal structure and axonal transport. Long peripheral axons depend on coordinated transport of mitochondria, vesicles, proteins, organelles, and neurotrophic signals mediated by kinesin and dynein.

Current mechanistic evidence increasingly supports distal axonal degeneration as a final common pathway of injury (29–32). Paclitaxel can produce early and sustained sensory axonal dysfunction (33). Experimental comparisons of eribulin, vincristine, paclitaxel, and ixabepilone have also demonstrated drug-specific effects on fast axonal transport and kinesin-driven microtubule movement (34).

These findings make impaired axonal transport a plausible mechanism of utidelone-associated neuropathy. Plausible, however, is not confirmed. The taxane literature is extensive and instructive, but utidelone has distinct binding kinetics, a different resistance profile, and is used in a more heavily pretreated population. Extrapolating mechanistic conclusions from paclitaxel to utidelone is scientifically reasonable as a starting point; treating it as established fact would be a clinical error.

### 5.2 Mitochondrial dysfunction and oxidative stress

Mitochondria support axonal energy production, calcium homeostasis, membrane potential, ion gradients, and repair processes. Taxane- and platinum-associated CIPN models demonstrate several abnormalities: mitochondrial swelling, impaired respiratory-chain function, reduced membrane potential, ATP depletion, and elevated reactive-oxygen-species generation (35).

Experimental models offer a clue: when researchers modulate mitochondrial electron transport, paclitaxel-induced pain changes. That does not prove mitochondria are the culprit in utidelone neuropathy, but it makes them a suspect worth watching (36).

Direct utidelone-specific evidence emerged in 2025. Gao and colleagues found that utidelone induced mechanical and cold allodynia in mice, accompanied by changes in oxidative stress-related proteins and increased TRPA1 expression in the dorsal root ganglia (28). Treatment with the TRPA1 antagonist HC-030031 and the antioxidants mito-TEMPO and edaravone reduced pain-like behavior.

This study provides direct utidelone-specific preclinical support for the involvement of oxidative stress and TRPA1 signaling in nociceptive hypersensitivity.

### 5.3 Neuroinflammation and glial signaling

Preclinical models demonstrate that chemotherapy may activate macrophages, satellite glial cells, Schwann cells and spinal glia. These cells may release inflammatory mediators such as tumor necrosis factor-α, interleukin-1β and interleukin-6 (37, 38).

Neuroinflammation may lower nociceptor thresholds, increase spontaneous neuronal activity, amplify pain transmission, impair nerve repair, and promote central sensitization. That said, no direct human studies have confirmed that utidelone activates these inflammatory pathways. Neuroinflammation therefore represents an extrapolated mechanism.

### 5.4 TRPA1-related signaling pathways

TRPA1 has been implicated in chemotherapy-associated mechanical hypersensitivity and cold allodynia. Experimental TRPA1 blockade can reduce persistent sensory neuropathy caused by neurotoxic chemotherapy (39). TRPA1 is also activated by oxidative stress products and inflammatory mediators (40). The utidelone mouse study provides additional support for TRPA1 involvement (28).

While well-validated clinical evidence specific to utidelone for TRPA1 is currently lacking, the established role of TRPA1 in chemotherapy-related mechanical and cold allodynia offers mechanistic hypotheses for studying pain phenotypes linked to utidelone. Future work could pursue utidelone-specific validation targeting cold allodynia, mechanical allodynia, oxidative stress, and TRPA1-related signaling pathways.

### 5.5 Schwann cell injury

Schwann cells contribute to myelin maintenance, axonal metabolic support and post-injury regeneration (41). Oxidative stress, inflammation and mitochondrial injury can all compromise their reparative capacity. Many patients treated with utidelone have prior taxane or platinum exposure; even with partial symptom resolution, peripheral nerve support systems may not fully recover. Re-exposure to microtubule-stabilizing agents can further reduce compensatory capacity, manifesting as worsening symptoms or delayed recovery.

### 5.6 Interindividual and genetic susceptibility

Substantial inter-individual variability exists in CIPN susceptibility. Gene polymorphisms related to drug transport, inflammation, mitochondrial function, DNA repair, and ion channels may all modify the risk of neurotoxicity (42).

Earlier studies reported associations between paclitaxel neuropathy and variants such as CYP2C8*3 (43) and identified candidate loci through genome-wide association studies (44). However, replication has been inconsistent.

A 2026 systematic review of 72 genetic association studies concluded that heterogeneous CIPN definitions, population bias, inconsistent statistical methods, and limited replication continue to prevent routine clinical translation of pharmacogenomic biomarkers (45). No genetic marker has been validated to predict utidelone-associated neuropathy.

## 6 Risk identification

### 6.1 Baseline characteristics and risk factors

Most patients treated with utidelone have a history of prior taxane chemotherapy, which defines the primary clinical population eligible for this regimen (1–3). This prior exposure is not merely a historical footnote. Even when taxane-induced symptoms have partially resolved, the underlying neural support system—Schwann cell repair capacity, axonal regenerative reserve, and microtubule dynamics—may not have fully recovered. Utidelone does not act on a pristine nervous system; it acts on one that is already depleted. This cumulative-injury framework is central to understanding why some patients deteriorate rapidly and recover slowly.

The 2025 multicenter real-world study found that unresolved peripheral neuropathy from previous therapy was associated with a greater likelihood of grade ≥3 neuropathy during utidelone treatment (6). Baseline assessment should therefore document preexisting symptoms and functional deficits—including distal paresthesia, neuropathic pain, vibration loss, reflex reduction, impaired hand function, gait instability, fall history, and any prior dose adjustments due to neuropathy. Other established risk factors for CIPN include advanced age, diabetes, obesity, renal insufficiency, nutritional deficiencies, alcohol use and concurrent neurotoxic medications, although their predictive strength varies across drug classes and populations (11, 31).

A 2026 cross-sectional study reported that age 60 years or older, a history of resolved neuropathy, and concurrent use of nonchemotherapeutic neurotoxic medications were independently associated with CIPN (46).

### 6.2 Potentially modifiable contributors

Clinicians should also screen for reversible contributors such as: poorly controlled diabetes; vitamin B12 deficiency; thyroid dysfunction; renal impairment; malnutrition; excessive alcohol use; potentially neurotoxic concomitant medications and environmental fall hazards.

Correcting these factors is appropriate general medical care. However, evidence that doing so prevents utidelone-associated neuropathy is unavailable.

### 6.3 Differential diagnosis

Atypical features during utidelone treatment include: marked asymmetry; dermatomal pain; prominent proximal weakness; rapidly progressive weakness; bowel or bladder dysfunction; cranial nerve abnormalities; upper motor neuron signs and progression unrelated to treatment exposure.

Potential alternative diagnoses include: diabetic neuropathy; radiculopathy; spinal stenosis; carpal or tarsal tunnel syndrome; vitamin deficiency; immune-mediated neuropathy; paraneoplastic neuropathy; leptomeningeal disease and metastatic spinal cord or nerve root compression.

## 7 Prevention and management

Neither the ESMO-EONS-EANO guideline nor the ASCO guideline recommends routine pharmacologic prophylaxis for CIPN (16, 17). In the absence of adequately powered utidelone-specific prevention trials, this principle should also apply to patients receiving utidelone.

**Table 2** stratifies management strategies by evidence group (Groups 1--5, as defined in Section 2.2), separating direct utidelone evidence from extrapolated CIPN data. Group 1 (direct comparative human) evidence is available for only electroacupuncture; most recommendations rely on Group 4 (extrapolated) or Group 5 (author-proposed) evidence. Benefits demonstrated in taxane-associated or mixed CIPN populations should not be interpreted as confirmation of efficacy for utidelone-associated neuropathy.

**Table 2:** Evidence-stratified management of utidelone-associated peripheral neuropathy.

Strategy	Evidence group	Evidence applicable to utidelone	Clinical position	Ref
Baseline and monitoring	G2, G4	Prior unresolved neuropathy linked to severe toxicity (G2); broader CIPN data support combined assessment (G4)	Routine care; intensify surveillance if baseline neuropathy present	(6, 16, 17, 19, 20)
Dose modification	G1,G5	Neuropathy is dose-limiting in trials; no randomized modification strategy	Interrupt, reduce, or stop per label if symptoms progress or function declines	(1, 6, 16, 17)
Education and rehabilitation	G4	No utidelone-specific trial; broader data support safety measures and exercise	Reasonable supportive care for functional impairment	(14–17, 47)
Duloxetine	G4	No utidelone-specific trial; RCTs show modest benefit in painful CIPN	For established painful CIPN only; not for numbness or prophylaxis	(17, 48)
Electro- acupuncture	G1, G4	One small utidelone RCT favored electroacupuncture over mecobalamin (G1); broader data suggest possible benefit (G4)	Optional adjunct; limited by small sample and no sham control	(49–53)
GM1	G2, G4	Secondary phase III analysis suggested lower burden (G2); broader data heterogeneous (G4)	Hypothesis-generating; not routine prophylaxis	(54–56)
Cryotherapy and Scrambler	G4	No utidelone evidence; data mainly from taxane or mixed CIPN	Investigational; not for routine utidelone use	(16, 17, 57–59)
Vitamin B12 and ALCAR	G4	Replace B12 only if deficient; ALCAR showed no benefit and possible harm in taxane patients	Do not use empirically; avoid ALCAR prophylaxis	(16, 17, 49, 60, 61)

ALCAR, acetyl-L-carnitine; CIPN, chemotherapy-induced peripheral neuropathy; GM1, monosialotetrahexosylganglioside; G1–G5, evidence groups 1–5 as defined in Section 2.2.

### 7.1 Patient education, safety and adjustment

Before treatment, patients should be informed about: numbness, tingling, neuropathic pain, cold and tactile hypersensitivity, impaired dexterity, unsteady gait, and elevated fall risk. Patients should also understand that dose modification, interruption, or discontinuation may be necessary during utidelone treatment. Clinicians should actively encourage early symptom reporting and reassure patients that disclosing discomfort will not automatically compromise their cancer treatment. Basic home safety strategies are fundamental supportive measures. These include routine hand and foot self-examination for unnoticed injuries, consistent use of supportive and well-fitted footwear, avoidance of barefoot walking, optimized indoor lighting, removal of household fall hazards, installation of handrails, use of protective gloves when handling hot or sharp objects, and seeking assistance when balance is impaired. While these low-cost interventions are clinically reasonable, current evidence does not confirm that they reduce the overall incidence of utidelone-related neuropathy.

Individualized dose adjustment is central to clinical management. CIPN-driven dose reduction inevitably raises concerns about compromised treatment intensity and oncologic efficacy. Reduced relative dose intensity correlates with inferior outcomes in early-stage breast cancer (62), but that relationship may not hold in late-line metastatic disease. No data define the minimum dose intensity required to sustain survival benefit with utidelone. Dose modification is not merely a safety reflex; it is a negotiation between tumor control and the patient’s ability to walk, write, and sleep. In late-line metastatic disease, preserving a few percentage points of response rate matters little if the patient loses the ability to live independently. Prior dose adjustments, potential reversibility, and patient preferences should also be taken into account. Mild, stable symptoms without functional impairment may permit continuation of utidelone, provided monitoring is intensified. However, progressive deficits, gait instability, recurrent falls, impaired self-care, or grade ≥2 neuropathy should trigger prompt reassessment of treatment intensity. Supportive measures and dose modification are not sequential steps—one does not exhaust the former before resorting to the latter. They should proceed in parallel. Waiting for acupuncture or GM1 to ‘work’ while a patient’s gait worsens is not cautious practice; it is a failure to recognize that no adjunct has been proven to halt progressive neurotoxicity.

### 7.2 Pharmacological and nutritional interventions

Duloxetine has the strongest randomized evidence for treating established painful CIPN. In a placebo-controlled trial, duloxetine produced a greater reduction in average pain and modestly improved pain-related function (48). ASCO identifies duloxetine as the pharmacologic treatment supported by the strongest evidence for painful CIPN (17). However, several limitations temper its broad use in utidelone-associated neuropathy: no utidelone-specific trial has been conducted, efficacy is limited to pain relief rather than sensory deficit improvement, benefit for numbness-dominant neuropathy is unproven, and adverse effects and drug interactions require individualized assessment. Common adverse effects include nausea, dizziness, somnolence, dry mouth, and blood pressure fluctuations. Caution is needed in patients with hepatic dysfunction and those receiving complex serotonergic regimens. Duloxetine should therefore be reserved for symptomatic painful CIPN rather than used for routine prophylaxis or universal management of all utidelone-induced neurological symptoms.

A secondary analysis of phase III utidelone trial data suggested that monosialotetrahexosylganglioside (GM1) was associated with a lower reported burden of peripheral neuropathy (54). However, GM1 use was not randomly assigned, and residual confounding and selection bias cannot be excluded. A meta-analysis across different chemotherapy regimens reported possible benefit but identified substantial clinical and methodological heterogeneity (55). Evidence from an earlier study in patients receiving oxaliplatin for gastrointestinal tumors cannot be directly generalized to utidelone-associated neuropathy (56).

Taken together, current evidence does not support routine GM1 prophylaxis for utidelone-treated patients. Clinical application, where considered, requires individualized discussion of uncertain efficacy, regulatory status, cost, and patient preferences.

Vitamin B12 plays important roles in methylation, myelin maintenance, and neurologic function (60), and therefore have biological plausibility as neuroprotective agents. However, empiric use in patients without documented deficiency has not been shown to prevent or treat CIPN. A 2025 randomized controlled trial comparing electroacupuncture with mecobalamin for established utidelone-associated neuropathy found superior symptomatic improvement with electroacupuncture at weeks 4 and 8 (49). Notably, mecobalamin served solely as a control intervention in this trial, so the data do not support combined use of electroacupuncture and mecobalamin. Clinically, mecobalamin supplementation is justified only for documented vitamin B12 deficiency; routine empiric use for utidelone-related CIPN is not supported.

A phase IIA trial suggested that acupuncture may reduce CIPN severity during weekly paclitaxel treatment in patients with breast cancer (50). A randomized trial comparing acupuncture with a sham procedure also reported improvement in selected CIPN symptoms (51).

The 2025 utidelone-specific randomized trial found that electroacupuncture produced greater improvement than mecobalamin in patient-reported sensory and motor symptoms, CTCAE grades, and selected quality-of-life domains (49). A 2025 systematic review and meta-analysis of 10 randomized trials involving 653 patients with breast cancer found that acupuncture improved overall clinical response, pain intensity, and neurotoxicity-specific scores. Subgroup analyses suggested possible benefit in both taxane- and utidelone-associated CIPN (52). A 2026 network meta-analysis of 32 randomized trials found that acupuncture-based interventions significantly reduced CIPN-related pain compared with controls (53). A multicenter randomized controlled trial found that exercise during chemotherapy reduced CIPN symptoms (47).

Programs should be individualized. Bone metastases, fracture risk, severe anemia, thrombocytopenia, active infection, uncontrolled pain, cardiopulmonary disease, or marked functional decline may require supervised or modified exercise.

Cryotherapy and limb compression therapy have been widely investigated for taxane-induced neuropathy but cannot be reliably generalized to utidelone treatment because of differences in infusion schedules, pharmacokinetics, cumulative exposure, patient populations, and neuropathy phenotypes (57). In addition, these physical interventions are poorly tolerated or contraindicated in patients with cold allodynia, Raynaud’s phenomenon, peripheral arterial disease, fragile skin, or diabetic foot complications, limiting their applicability in utidelone-treated cohorts. Both approaches should remain investigational.

Scrambler therapy is a noninvasive neuromodulation approach that relieves neuropathic pain by replacing pathological nociceptive signals with nonpainful sensory input. Pilot studies have demonstrated symptomatic improvement in CIPN patients, and phase II trial data comparing Scrambler therapy with transcutaneous electrical nerve stimulation provide preliminary supportive evidence (58, 59).

However, widespread application is limited by small sample sizes, inconsistent protocols, blinding challenges, uncertain durability of effect, and limited clinical accessibility. Scrambler therapy may be considered as a salvage intervention for refractory painful CIPN after first-line options have been exhausted, but it should not be regarded as routine first-line management.

Acetyl-L-carnitine should not be used routinely for CIPN prevention. A randomized, placebo-controlled trial in women receiving taxane therapy failed to demonstrate preventive benefit and raised concern about worsening neuropathy during longer-term follow-up (61).

Both major international guidelines advise against the routine use of acetyl-L-carnitine for CIPN prevention (16, 17).

## 8 Proposed full-cycle clinical workflow

**Figure 2:**
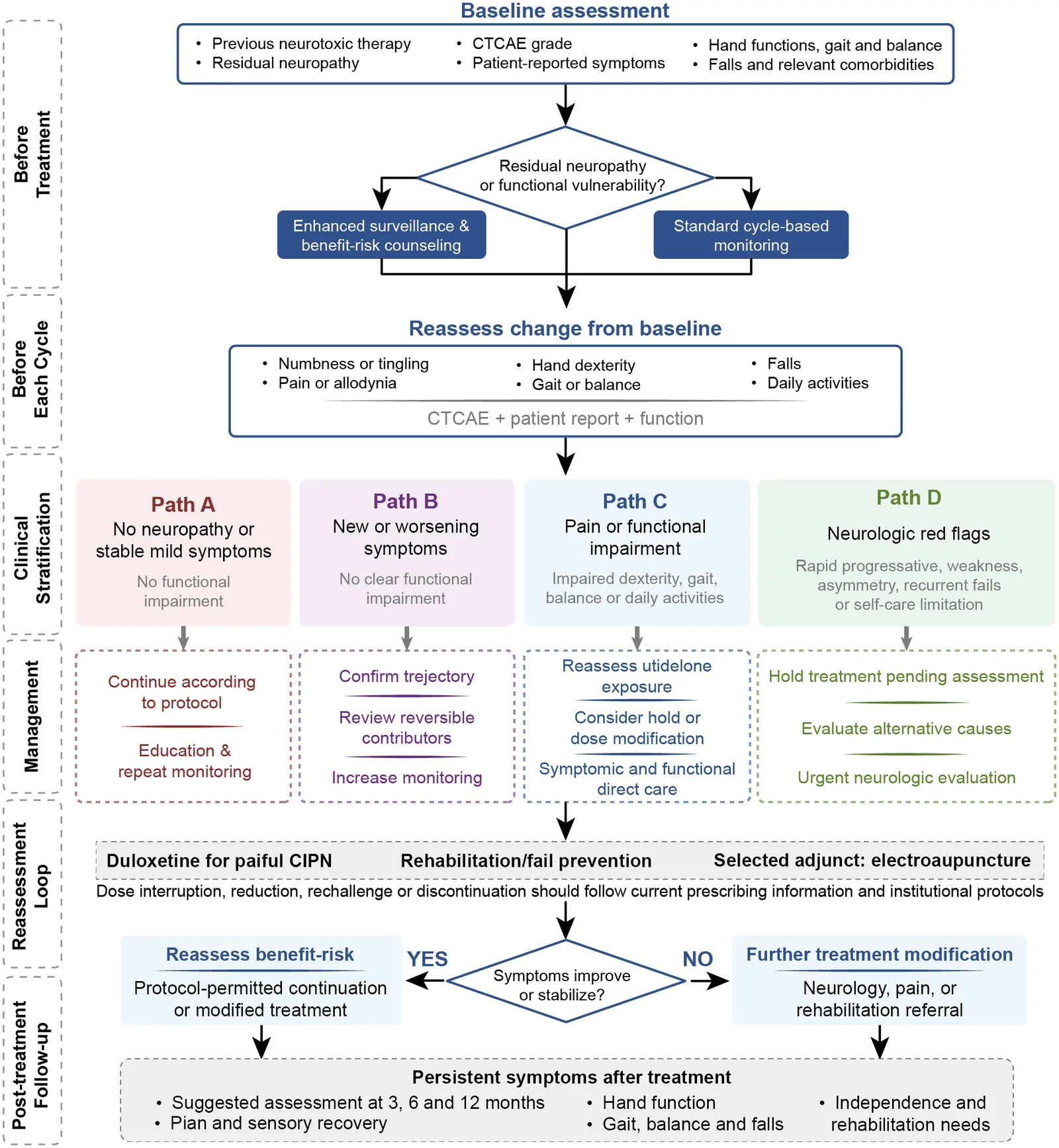
Full-cycle monitoring and management framework for peripheral neuropathy during utidelone treatment, stratified by evidence group. Groups 1-2 (direct human evidence): Baseline risk factors and treatment-limiting toxicity data from utidelone trials inform pretreatment assessment and dose-modification thresholds. Group 4 (extrapolated): Assessment instruments, fall-prevention strategies, and rehabilitation approaches are adapted from broader CIPN guidelines. Group 5 (author-proposed): The specific cycle-by-cycle escalation sequence, the 3-/6-/12-month follow-up schedule, and the integration of patient-reported outcomes with functional evaluation represent the authors’ clinical practice, pending prospective validation. This workflow has not been prospectively validated (6, 16, 17, 19, 20, 47–49, 63).

**Figure 2** presents a full-cycle framework proposed by the authors. It integrates pretreatment assessment, cycle-by-cycle monitoring, symptom- and function-based escalation, reassessment after intervention or treatment modification, and post-treatment follow-up. The framework incorporates direct utidelone evidence, international CIPN guidelines, and evidence extrapolated from broader CIPN populations.

### 8.1 Before treatment

Before treatment, clinicians should document prior neurotoxic exposure, unresolved neuropathy, baseline CTCAE grade, patient-reported symptoms, neurologic and functional findings, fall history, and clinically relevant comorbidities or concomitant medications. Patients with residual neuropathy or functional vulnerability require enhanced surveillance and explicit benefit-risk counseling (6, 16, 17, 19, 20).

### 8.2 Before every treatment cycle

Before each cycle, clinicians should assess changes from baseline in sensory symptoms, pain, hand dexterity, gait, balance, falls, sleep, and daily activities (16, 17, 19, 20). Remote monitoring may supplement but should not replace clinical assessment; alert thresholds and escalation responsibilities should be predefined.

### 8.3 Escalation based on symptoms and function

Management intensity should be guided by symptom trajectory, functional impact, fall risk, anticipated anticancer benefit, available alternatives, and patient preference. If a patient has no symptoms, or only mild tingling that does not interfere with daily life, continuing utidelone is reasonable—but only if the patient knows exactly what to watch for and when to call. Reinforced education is not a passive default; it is an active safety measure. When new or worsening symptoms develop without clear functional impairment, clinicians should verify the symptom trajectory, intensify monitoring, and review potentially reversible contributors or alternative diagnoses. If neuropathy causes pain requiring treatment, impaired dexterity, gait instability, falls, or interference with daily activities, reassess utidelone tolerability and consider dose interruption or reduction per the prescribing information. Mild, stable symptoms without functional impairment may permit continuation of utidelone, provided monitoring is intensified. However, progressive deficits, gait instability, recurrent falls, impaired self-care, or grade ≥2 neuropathy should trigger prompt reassessment of treatment intensity. Supportive measures alone should not justify maintaining full-dose utidelone while neurologic function deteriorates; dose holds, reductions, or discontinuation should follow product labeling and institutional protocols. For painful CIPN, duloxetine may be added if no contraindications exist (17, 48). Exercise and rehabilitation do not reverse the underlying neural injury, and evidence that they reduce CIPN incidence is mixed. Their value lies elsewhere: preserving muscle strength, retraining balance, preventing falls, and maintaining the patient’s confidence in mobility. For a patient with bone metastases, a single fall can be catastrophic; in this context, rehabilitation is not an optional adjunct but a safety imperative. Programs must be individualized—bone lesions, anemia, thrombocytopenia, and cardiopulmonary status all modify what is safe (14, 15, 47). Electroacupuncture may be an adjunct in selected patients (49).

When severe functional loss, recurrent falls, self-care deficit, rapidly progressive weakness, asymmetry, bowel or bladder dysfunction, cranial nerve signs, or upper motor neuron findings are present, urgent evaluation for other neurologic conditions is needed. Utidelone should not be continued at full dose if neurologic function is deteriorating (16, 17).

### 8.4 Multidisciplinary coordination

Effective management of utidelone-associated neuropathy rarely falls within a single specialty. In our practice, medical oncologists retain responsibility for balancing anticancer efficacy against neurotoxicity and for dose-modification decisions. Nursing staff conduct cycle-by-cycle symptom screening and PRO collection. Neurology consultants assist when the clinical picture is atypical, rapidly progressive, or diagnostically uncertain, and they perform nerve conduction studies when indicated. Pain specialists manage refractory neuropathic pain that does not respond to duloxetine. Rehabilitation medicine prescribes individualized exercise, balance training, and fall-prevention programs. Acupuncture, when offered, is delivered by certified practitioners with explicit understanding that it is an adjunct, not a replacement for dose modification. Nutritionists address malnutrition, vitamin deficiencies, and low albumin that may impair neural repair. This division of labor is not merely administrative; it ensures that no single clinician bears the impossible task of optimizing both tumor control and neural function alone.

### 8.5 Follow-up

Assess pain, sensory recovery, hand function, gait, balance, falls, sleep, self-care, rehabilitation needs, and use of analgesics or assistive devices at 3, 6, and 12 months after treatment. Long-term follow-up is supported by evidence that neuropathy can persist beyond active treatment (63).

## 9 What is known, what is extrapolated, and what remains unknown

**Table 3** provides an evidence map that distinguishes conclusions directly supported by utidelone-specific research from those extrapolated from the broader CIPN literature and identifies the principal remaining knowledge gaps.

**Table 3:** Evidence map and key gaps in utidelone-associated peripheral neuropathy.

Domain	Directly supported (G1–G3)	Extrapolated or unknown (G4–G5)	Ref
Incidence	Any-grade neuropathy 55–59% in pivotal and real-world studies; grade 3 22% in phase III	Standardized incidence across regimens and tumors	(1, 6–8)
Schedule and risk factors	Prior unresolved neuropathy and days 1–5 schedule linked to severe toxicity	Independent predictors; whether continuous infusion reduces risk	(6, 8)
Phenotype	Clinically important, often sensory	Onset, cumulative-dose relationship, fiber type, persistence, recovery	(1, 6, 9–15, 18, 63)
Assessment	No utidelone-specific instrument validated	Optimal combination of CTCAE, PROs, and objective measures	(16, 17, 19–27)
Mechanisms	Microtubule stabilization established; mouse data support oxidative stress/TRPA1 allodynia	Roles of axonal transport, mitochondrial injury, inflammation, Schwann cell damage in humans	(1, 3, 28–41)
Biomarkers	None validated	Clinical, genetic, and multi-omic prediction models	(42–46)
Prevention	GM1 secondary analysis suggested lower burden	Effective prevention without compromising efficacy	(54–57, 61)
Treatment	One small RCT favored electroacupuncture; no utidelone duloxetine or rehab trial	Comparative efficacy for pain, function, and long-term recovery	(17, 47–53, 58, 59)

CIPN, chemotherapy-induced peripheral neuropathy; CTCAE, Common Terminology Criteria for Adverse Events; GM1, monosialotetrahexosylganglioside; G1–G5, evidence groups 1–5 per Section 2.2; PRO, patient-reported outcome; TRPA1, transient receptor potential ankyrin 1.

The evidence is strongest for incidence and clinical burden but remains limited regarding phenotype, natural history, risk prediction, human mechanisms, prevention, and recovery.

## 10 Future research directions

Future work should shift from passive adverse-event reporting to prospective risk prediction, mechanistic validation, and interventional evaluation with structured long-term follow-up.

Clinical characterization and risk prediction. Multicenter prospective cohorts should designate CIPN as a primary or key secondary endpoint, using a standardized battery (CTCAE, EORTC QLQ-CIPN20 or FACT/GOG-Ntx, PRO-CTCAE, pain scores, and functional measures such as vibration sensation, grip strength, gait, and balance) at baseline, every 1–2 cycles, at treatment completion, and at 3, 6, and 12 months post-therapy. Analyses should stratify by clinical phenotype—painful, sensory-dominant, motor, or balance-related—to guide phenotype-specific management (16, 17, 19–27, 63).

Risk models should integrate age, performance status, BMI, diabetes, renal function, nutritional status and albumin, prior taxane or platinum exposure, baseline neuropathy, cumulative utidelone dose, initial CTCAE/PRO scores, and electrophysiologic parameters. Development must be followed by external validation, calibration, and assessment of clinical net benefit, not merely discrimination (6, 11, 27, 31, 42–46).

Interventional trials. Randomized studies should evaluate primary prevention, early intervention, and treatment of established neuropathy in high-risk patients (16, 17, 47–61).

Mechanistic research. Priority targets include microtubule dynamics, axonal transport, mitochondrial function, neuroinflammation, Schwann cell repair, TRPA1/Nav channel signaling, and axonal regeneration (28–33, 35–41).

Patient-centered outcomes. Sleep, mood, falls, work capacity, activities of daily living, caregiver burden, treatment satisfaction, and financial toxicity should be formally incorporated. Interventions that preserve function, prevent falls, or maintain independence warrant evaluation even if they do not reduce overall CIPN incidence (13–15, 19, 20).

## 11 Limitations

We acknowledge the limitations of this narrative approach. We did not register a protocol, perform dual independent screening, or apply formal risk-of-bias tools, because our primary aim was to map the applicability of available evidence to utidelone rather than to synthesize effect sizes. The evidence categories we used—distinguishing direct utidelone data from extrapolated findings—reflect clinical relevance, not formal certainty grades.

The utidelone-specific evidence chain is fragmentary. Nearly all available data come from adverse-event tabulations in the pivotal phase III trial and subsequent observational reports, not from studies designed to evaluate CIPN as a primary endpoint. We know the incidence rates, but we do not know the median time to symptom onset, the cumulative dose-toxicity relationship, the relative frequency of painful versus numbness-dominant phenotypes, or the probability of recovery after stopping treatment. Extrapolation from taxane and platinum literature provides biological plausibility, yet it cannot confirm which mechanisms operate in utidelone-treated humans or which interventions actually change the clinical course.

Outcome measurement is another source of uncertainty. CIPN is not a single entity: it may present as sensory loss, burning pain, gait instability, or impaired hand function. Yet studies have mixed CTCAE grades, nerve conduction data, pain scores, EORTC QLQ-CIPN20, FACT/GOG-Ntx, and PRO-CTCAE items without standardized timing or thresholds. A patient may report life-altering symptoms while the CTCAE box still says grade 1. This methodological heterogeneity is not a trivial statistical nuisance; it is a primary reason why meta-analyses of CIPN interventions yield fragile conclusions and why guideline recommendations remain weak.

Consequently, the framework we propose is a pragmatic starting point, not a validated guideline. We do not know which patients should receive preventive intervention, whether GM1 or acupuncture alters the trajectory of utidelone neuropathy, or how many patients recover neurologic function at 6 or 12 months after the last dose. Until prospective, longitudinal studies with standardized patient-reported and functional outcomes become available, clinicians must manage utidelone-associated neuropathy with incomplete information. The goal of this review is to make that uncertainty explicit, not to disguise it.

## 12 Conclusion

Utidelone provides a clinically important survival option for patients with metastatic breast cancer after anthracycline and taxane failure, yet peripheral neuropathy remains its dominant dose-limiting toxicity. In the pivotal phase III trial and large real-world series, more than half of patients developed treatment-emergent neuropathy, with grade 3 events in roughly one in five. Prior unresolved neurotoxicity appears to heighten susceptibility, so documenting baseline sensory and functional status before the first dose is essential.

The mechanistic picture is incomplete. The only direct data tie utidelone-induced pain-like behavior to TRPA1 upregulation and oxidative stress in the dorsal root ganglia; whether extrapolated pathways from taxane and platinum models–such as axonal transport failure or mitochondrial injury–explain the numbness-dominant phenotype, gait instability, or persistent deficits seen clinically remains entirely unknown.

This uncertainty mandates a cautious, function-centered approach. Routine pharmacologic prophylaxis is not supported by current guidelines or by direct utidelone evidence. Duloxetine may help painful symptoms, and electroacupuncture shows promise as an adjunct, but neither should delay dose modification when function declines. The suggestion that GM1 might reduce neuropathy burden comes from a secondary analysis and remains hypothesis-generating; it cannot be recommended as standard prophylaxis.

The need is prospective, longitudinal research that follows patients from baseline through treatment and recovery with standardized symptom and functional measures. We still do not know who is most vulnerable, whether schedule modifications materially reduce risk, or which interventions protect nerve function without compromising anticancer efficacy. Until such data emerge, clinicians should rely on pretreatment risk assessment, cycle-by-cycle monitoring of patient-reported symptoms and physical function, and timely modification of utidelone exposure when neurologic injury threatens independence. The goal is not merely to record toxicity, but to preserve the patient’s ability to walk, work, and live without fear of falling.
